# Clinical characteristics and management of 106 patients with pyogenic liver abscess in a traditional Chinese hospital

**DOI:** 10.3389/fsurg.2022.1041746

**Published:** 2023-01-06

**Authors:** ShiJiao Nie, Da Lin, XiaoWen Li

**Affiliations:** ^1^ Department of Hospital Infection Management, Hangzhou First People's Hospital, Zhejiang University School of Medicine, Hangzhou, China; ^2^ Department of General Surgery, Hangzhou TCM Hospital Affiliated to Zhejiang Chinese Medical University, Hangzhou, China

**Keywords:** pyogenic liver abscess, clinical features, pathogenic bacteria, outcomes, laboratory features

## Abstract

This is a retrospective study of clinical data from 106 patients with pyogenic liver abscess (PLA) treated in a traditional Chinese hospital during the eight years preceding this publication. We aimed to provide evidence to improve the diagnosis accuracy and the treatment strategies for PLAs. We collected records of patients treated at the Guangxing Hospital, which is affiliated to the Zhejiang Traditional Chinese University in Hangzhou, and we collected their general background information, laboratory and imaging features, and clinical manifestations and outcomes to perform a retrospective analysis. Diabetes mellitus (45.3%, 48/106), biliary calculi (36.8%, 39/106), and history of abdominal surgery (15.1%, 16/106) were the three most common PLA risk factors present in our cohort. Fever and chills (95.3%, 101/106), right upper quadrant pain/epigastric discomfort (68.9%, 73/106), nausea and vomiting (38.8%, 41/106), and cough and sputum (14.2%, 15/106) were the most common clinical manifestations of PLA. Most patients had the abscesses in the right liver lobe, and the most commonly found bacteria were *Klebsiella pneumoniae* (54.8%, 42/76), *Escherichia coli* (35.1%, 27/76), and *Streptococcus pneumoniae* (3.9%, 3/76). Liver Doppler ultrasound is a conventional and effective method to identify liver abscesses. Most patients were treated using a percutaneous puncture under B-ultrasound guidance. Most patients (*n* = 104 or 98.1%) were cured, one patient (0.9%) died, and one was discharged with multiple abscesses post treatment.

## Introduction

Pyogenic liver abscesses (PLAs) are reported globally, but their incidence varies significantly between countries (from 8 to 22 patients per 1,000,000 people) (1). The incidences of PLA in Asia are higher than those in western countries, with the highest being in Taiwan, China (2, 3). Medical technological progress has reduced the PLA mortality risk. However, regional differences in the application of these technologies have caused the mortality rate to remain high. A review by Chan KS et al. of 16 PLA articles showed mortality rates ranging from 0% to 15.7% (11 articles) (4).

In the early 20th century, the most common route of PLA infection was portal phlebitis, often secondary to acute appendicitis, and the total mortality rate was as high as 80% (5). However, the different infection routes described since then include biliary calculi, the portal vein, hepatic artery, cryptogenic pathway, and adjacent infections (6). Some studies have reported diabetes mellitus as a risk factor for PLA (2). Recent studies have shown that biliary tract alterations (including acute cholecystitis, common bile duct stones, chronic pancreatitis, and tumoral biliary obstructions) are the main route of infection for PLA (7, 8). The number of cryptogenic liver abscess cases has been on the rise, and Thomas McNeil et al. found no predisposing conditions (cryptogenic) for 14 (18%) of the patients in their cohort (9).

The clinical manifestations of PLA are nonspecific. The main symptoms are fever and chills, and there may be right upper quadrant pain/epigastric discomfort, but sometimes the signs are unclear. Early PLA diagnoses depend mainly on imaging examinations. B-ultrasound is the first choice, followed by abdominal computed tomography (CT) and magnetic resonance imaging (MRI). The risk factors for PLA include age, male sex, diabetes mellitus, biliary calculi, immunodeficiency, and the use of proton pump inhibitors (1, 10). And, the pathogens associated with PLA are mostly gram-negative bacilli, but reports of mixed infections of *Streptococci* and anaerobic bacteria exist.

The diagnosis and management of PLA have changed substantially in the past few decades. Due to the advent of advanced imaging techniques and accurate chemical tests, early diagnosis, precise localization, image-guided percutaneous aspiration, and drainage of abscesses can be achieved easily. However, many patients are missed due to the nonspecific clinical features of PLA, and a high degree of suspicion is the cornerstone of misdiagnosis prevention and prognosis improvement. We present here a retrospective analysis of 106 patients with PLAs managed in a Chinese hospital over the course of the eight years preceding the publication of this study. We analyzed demographic, clinical, laboratory, pathogenic, and management outcomes.

## Materials and methods

We obtained the medical records of inpatients with a principal diagnosis of a liver abscess between June 2013 and June 2021 from the clinical document database of the Guangxing Hospital affiliated to the Zhejiang Traditional Chinese University in Hangzhou. All the patients were diagnosed as having liver abscesses, and all were adults older than 18 years. The data could be found in Supplementary materials.

PLA was diagnosed if: (1) the results of microbial cultures (blood or pus cultures) were positive, or antimicrobial treatment was effective; and/or, (2) after percutaneous liver puncture or surgical treatment, a lesion with suppurative infection caused by bacteria was confirmed; and, (3) clinical manifestations such as fever, liver discomfort, abdominal pain, and percussion pain in liver area were present; and/or, (4) hepatic abscesses or lesions were diagnosed using liver Doppler ultrasound, CT, or MR images.

To decrease biases, we excluded patients with liver liquefaction infarctions, hepatic echinococcosis, amebic liver abscesses, tuberculous liver abscesses, and liver abscesses caused by other factors; patients lacking clinical data; and patients who had undergone radiofrequency ablation (RFA), transcatheter arterial chemoembolization (TACE), or who had had postoperative hepatic tumor-associated infections.

We extracted the following demographic data and clinical information from each patient's record: age, sex, presenting symptoms and signs, comorbidities, the imaging modality they had been diagnosed with, the abscess characteristics (size, location, and number of abscesses), whether percutaneous aspiration was performed, whether a drainage catheter was inserted, the hospital stay duration, and the treatment outcome. We retrieved the following laboratory variables for analysis: white blood cell count (WBC); neutrophil/leukocyte ratio; platelet count; serum levels of C-reactive protein (CRP), procalcitonin (PCT), albumin, total bilirubin, alkaline phosphatase (ALP), fibrinogen, and alanine transaminase (ALT), and creatine kinase (CK); and prothrombin time (PT).

All measurement data were normally distributed and we expressed them as means ± SDs. We expressed count data as frequencies (percentages).

## Results

### Basic features

During the period from June 2013 to June 2021, 106 patients were diagnosed as having PLA at the Zhejiang Chinese Medical University. We analyzed data from all the patients with available and complete records. The data belonged to 71 men and 35 women (male-to-female ratio, 2.03:1) with an age range between 18 and 87 years (mean ± SD, 64 ± 13 years). The age group with the most patients was the 60–69-year-old group, followed by the 70–79-year-old group.

### Clinical manifestations

As shown in Table 1, the most common presenting features were fever and chills in 101 patients (95.3%), followed by right upper quadrant pain in 73 patients (68.9%), nausea and vomiting in 41 patients (38.8%), cough and sputum in 15 patients (14.2%), and scleral icterus/yellow urine in 6 patients (5.7%). High fever (>39°C) was present in 77 patients (74.8%) on admission. The body temperatures ranged from 36.3°C to 41.3°C (mean ± SD, 39.3 ± 0.9°C).

**Table 1:** Clinical features of PLA.

Clinical features	Number of patients (%)
Fever (≥37.5°C) and chills	101 (95.3%)
High Fever (≥39°C)	77 (74.8%)
Nausea and vomiting	41 (38.8%)
Cough and sputum	15 (14.2%)
Scleral icterus/yellow urine	6 (5.7%)
Right upper quadrant pain/epigastric discomfort	73 (68.9%)
Diabetes mellitus	48 (45.3%)
Biliary calculi	39 (36.8%)
History of abdominal surgery	16 (15.1%)
Hepatic cyst	15 (14.2%)
Shock	15 (14.2%)
Gastrointestinal tumor	5 (4.7%)
Endophthalmitis	1 (0.94%)

The most common comorbidities included diabetes mellitus (48 patients, 45.3%), and biliary calculi (39 patients, 36.8%). We found 16 patients (15.1%) with a history of abdominal surgeries, 15 (14.2%) with hepatic cysts, and 2 (1.9%) with hepatic cysts complicated with bacterial infections. Our cohort included 15 patients (14.2%) with septic shock and 5 patients (4.7%) with gastrointestinal tumors. One patient, had had three different hepatic abscesses at different times without other intestinal lesions on contrast-enhanced CT images, but with a malignant tumor in the hepatic flexure of the colon (as seen during a colonoscopy). Another patient who felt eye discomfort after two weeks was diagnosed with endophthalmitis with Klebsiella pneumoniae being found in both the aqueous and pus cultures. Other comorbidities in our cohort included hypertension, senile stroke, ischemic heart disease, congestive heart failure, and malignant diseases.

### Laboratory features

The most common laboratory abnormality in our cohort of patients was CRP elevation in 105 of 106 patients (99.1%; Table 2), followed by decreased albumin levels in 98 of 103 patients (95.1%), elevated procalcitonin (PCT) levels in 71 of 85 patients (83.5%), elevated neutrophil/leukocyte ratio in 87 of 106 patients (82.1%), and abnormal WBCs in 74 of 106 patients (72.6%). We found high leukocyte counts in 71 patients (67.0%) and low counts in 3 patients (2.8%). Notably, infection-induced thrombocytopenia had occurred in 18 patients (17.0%), while we found elevated levels of ALP in 56 patients (52.8%), ALT in 54 patients (50.9%), and total bilirubin in 20 patients (18.9%). These last patients had mild to moderate liver function impairments. Additionally, 98 patients (92.5%) had elevated fibrinogen levels, 28 (26.4%) had elevated PTs, and 17 (16.0%) had high CK levels.

**Table 2:** Laboratory features of PLA.

Abnormal Laboratory Values	Number of patients (%)	Mean ± SD	Range
Albumin (high)	98/103 (95.1%)	32.4 ± 5.2	21.1–48.9
CRP (high)	105 /106 (99.1%)	161.3 ± 81.9	5.63–313
Neutrophil/leukocyte ratio	87/106 (82.1%)	82.9 ± 8.0	62.5–96.8
PCT (high)	71/85 (83.5%)	16.40 ± 30.48	0.04–100
WBC (abnormal)	74/106 (69.8%)	12.24 ± 5.21	0.98–32.46
WBC (high)	71/106 (67.0%)		
WBC (low)	3/106 (2.8%)		
Infection-induced thrombocytopenia	18/106 (17.0%)	81.9 ± 18.3	51–109
ALP (high)	56/106 (52.8%)	151.8 ± 98.0	47–687
ALT (high)	54/106 (50.9%)	61.5 ± 47.3	11–295
Bilirubin (high)	20/106 (18.9%)	19.2 ± 12.1	5.3–70.2
Fibrinogen (high)	98/106 (92.5%)	5.90 ± 1.61	2.93–13.19
PT (high)	28/106 (26.4%)	13.2 ± 1.6	9.7–17.8
CK (high)	17/106 (16.0%)	117 ± 133	12–747

CRP, C-reactive protein; WBC, white blood cell; PCT, procalcitonin; ALP, alkaline phosphatase; ALT, alanine transaminase; PT, prothrombin time; CK, creatine kinase.

The hemocultures or liver abscess fluid cultures of 76 patients were positive (Table 3) with 42 cases of *K. pneumoniae* (the most common pathogen in our cohort), 27 of *Escherichia coli*, 3 of *Streptococcus pneumoniae*, 2 of *Streptococcus intermedius,* 1 of *Lactococcus lactis* subspecies, 1 of *Enterobacter cloacae*, and 1 of *Citrobacter braakii*.

**Table 3:** Results of hemocultures or liver abscess fluid cultures of patients with PLA.

Pathogen	Number of patients (%)
*Klebsiella pneumoniae*	42 (54.5%)
*Escherichia coli*	27 (35.1%)
*Streptococcus pneumoniae*	3 (3.9%)
*Streptococcus intermedius*	2 (2.6%)
*Lactococcus lactis* subspecies	1 (1.3%)
*Enterobacter cloacae*	1 (1.3%)
*Citrobacter braakii*	1 (1.3%)

### Imaging features

Ultrasound alone was the diagnostic tool of choice in 62 patients (58.5%). CT scans or MRI were performed as appropriate for differential diagnoses of liver cancer or cancer with infections. The abscess diameters ranged from 1 to 12.7 cm (mean ± SD, 5.6 ± 2.4 cm). The PLAs were confined mostly to the right hepatic lobe (66 patients, 62.3%) due to the portal vein anatomy, high hepatic mass, and dense network of bile canaliculi in this lobe. We found 27 patients (26.2%) with PLAs in the left hepatic lobe. The PLAs in 13 patients (12.3%) were either in the right lobe, or in the right and left lobes, while two patients had PLAs in the caudate lobe. We found single abscesses in 83 patients (78.3%), and multiple abscesses in 23 patients (21.7%) (Table 4).

**Table 4:** Location and number of PLA.

Location	Number of patients (%)
Left lobe	27 (26.2%)
Right lobe	66 (62.3%)
Left and right lobes	13 (12.3%)
Single abscess	83 (78.3%)
Multiple abscesses	23 (21.7%)

### Treatment

**Figure 1:**
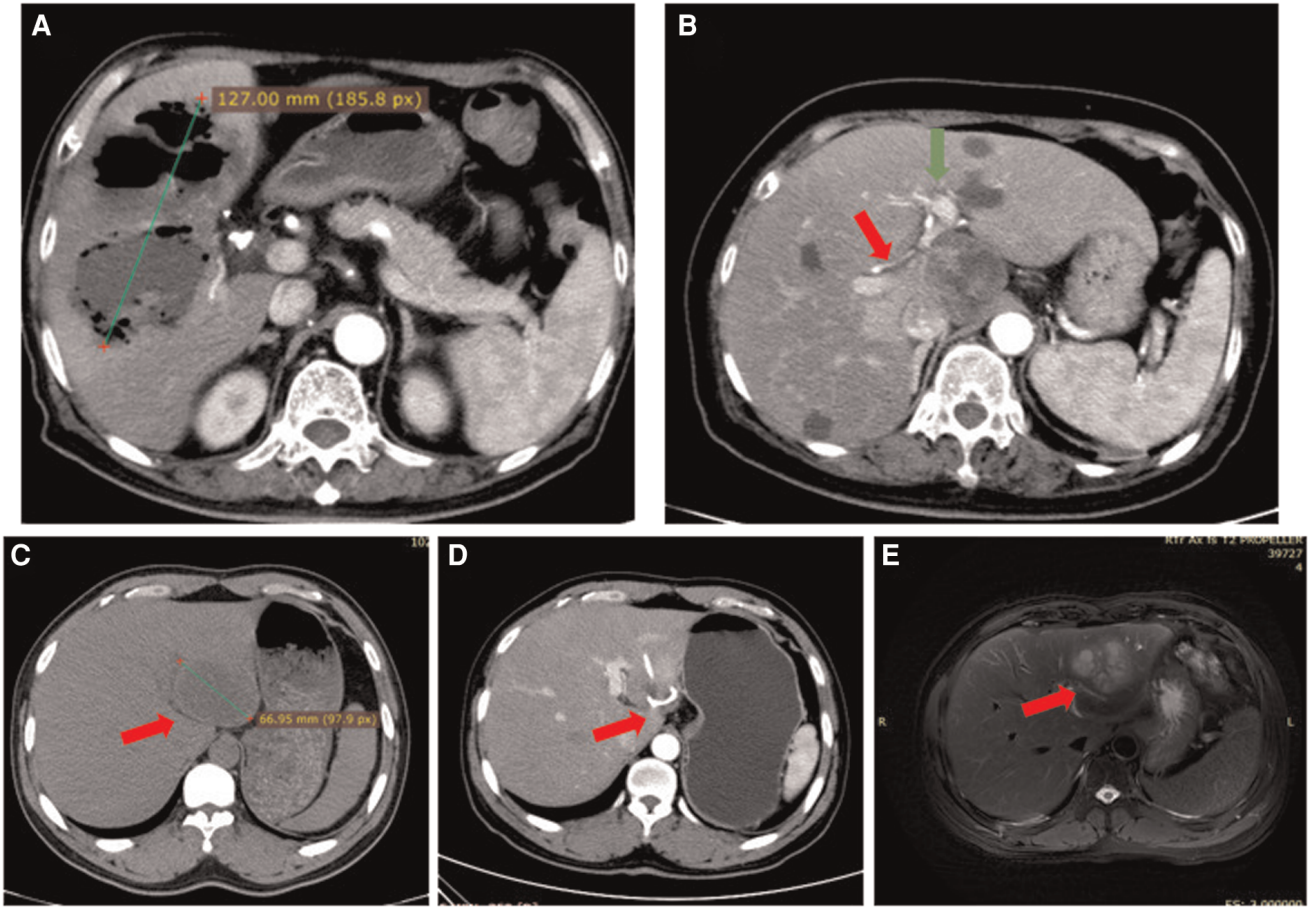
(**A**) case #1. Irregular low-density shadows were observed in the liver with clear boundaries and uneven internal density. Scattered gas density shadows were observed, and some gas-liquid planes were observed with a large cross section of approximately 127 mm. (**B**) Case #2. Image showing the lack of a space to place a drainage tube; The red arrow points to the right hepatic artery, The green arrow points to the left hepatic artery. (**C**) Case #3. The left lobe of the liver is a round low-density focus, approximately 53 mm in size. (**D**) Case #3. A drainage duct shadow was seen in the left lobe of the liver, with a few adjacent patchy low-density shadows. (**E**) Case #3. Recurrent abscess after treatment of liver abscess: A multilocular cystic solid long T2 signal shadow was seen in the left lobe of the liver, the maximum cross-section was 66 mm, and the boundaries were blurred. Left lobe lesion of the liver, abscess considered.

The 106 patients in our cohort received anti-infection therapy. In addition, those with liver damage received liver protection therapy, and those with septic shock received rapid rehydration and anticoagulation therapy. Under the guidance of B-ultrasound, 76 patients underwent puncturing and draining of abscesses with either one or more drainage tubes inserted; however, one patient had to undergo continued percutaneous aspiration due to lack of an appropriate drainage tube insertion space (Figure 1). Nine patients (8.5%) were treated with piperacillin sodium and tazobactam sodium, 42 (39.6%) with third generation cephalosporins combined with quinolones/nitrazole antibiotics, and 28 (26.4%) with third generation cephalosporins alone. In 27 patients (25.5%), the infections were controlled with the initial use of carbapenems, and the subsequent use of third generation cephalosporins and other antibiotics. Thirty patients (28.3%) were treated with anti-infectives alone, 76 (71.7%) were treated with ultrasound-guided puncture and drainage, and no one required surgical resection.

### Outcome

The patients' hospital stays lasted between 3 and 43 days, with the average length of stay at 19.9 ± 10.2 days. A total of 104 patients (98.2%) were cured. Despite careful treatment, an elderly patient with severe hypertension, diabetes mellitus, heart failure, and hypoproteinemia died. The mortality rate was 0.9%. Another patient, whose abscess was treated with ultrasound-guided puncture and drainage and who had a normal body temperature and hemogram, developed multiple small abscesses near the primary abscess as observed by CT and MRI techniques two weeks later (Figure 1).

## Discussion

Hippocrates described the first liver abscess in the year 400 BC. PLA remains a major public health problem due to its severe morbidity and mortality. PLA is an infective disease caused by various pathogens, and it often occurs in patients with impaired immunity such as those with diabetes mellitus and malignancies (11–13). In this study, we reviewed and analyzed the background features, clinical manifestations, laboratory and imaging features, and outcomes of 106 patients with PLA. One patient with multiple organ disease died*,* and one patient whose abscess was spread after treatment requested to be discharged.

The average age of the patients with PLA in our study was 64 years. This supports the findings of other studies indicating that the risk of PLA increases with age (14, 15). Also, our cohort had mostly men.

In patients with liver abscesses, large numbers of bacteria and toxins from the purulent cavity cause systemic sepsis with symptoms such as chills and fever. The presenting symptoms of PLAs are multiple and nonspecific, and include fever, right upper abdominal pain, vomiting, nausea, and asthenia. Fever and pain in the right upper abdomen have been found to be the main clinical symptoms (1, 15, 16). However, symptoms such as nausea and vomiting, cough and sputum, and the yellowing of the eyes have also been observed. In our study, 101 patients (95.3%) had fever [77 patients (78.7%) had mainly high fever]; while other common clinical manifestations included right upper quadrant pain (73 patients, 68.9%), nausea and vomiting (41 patients, 38.8%), and cough and sputum (15 patients, 14.2%). Atypical clinical symptoms (mostly in middle-aged and elderly patients with relatively slow responses) included sensitivity to pain combined with basic diseases generating discomfort such as abdominal pain; and, weakness, especially in patients with diabetes mellitus and visceral autonomic dysfunction, with an increased pain threshold (17, 18).

Comorbid illnesses included biliary calculi, diabetes mellitus, history of abdominal surgery, hepatic cysts, colon tumor, and endophthalmitis. Biliary calculi and diabetes mellitus were the predominant causes of PLAs (19). Biliary calculi were present in most patients (55%) of a different study (1), and yet another study reported coexistence rates for biliary calculi of 40.95%. The recurrence rates for patients with biliary PLAs have been recorded at percentages between 23% and 37%, and between 2% and 4% for those without biliary PLAs (20). This high recurrence risk may be clinically targeted; thus, patients with biliary PLAs need special attention. Diabetes mellitus is a risk factor for PLAs with a hazard risk rate ranging from 3.6 to 9-fold (21), and the disease is relatively common in patients with PLA; the reported coexistence rates are 30% in Hong Kong, 31% in Canada, 28.7% in a single center in Xi'an (China), 23% in Italy (1, 22), and 45.3% in our study. The morbidity of patients with PLAs and diabetes is high, and this may be due to their impaired immunity, neutrophil chemotaxis, mononuclear phagocyte activation, and/or opsonization. In addition, hyperglycemia can promote bacterial growth in tissues; and, metabolic disorders impact the liver, gut, pancreas, stomach, and intestine (14). Cryptogenic liver abscesses may be associated with gastrointestinal malignancies (23); and, colonoscopies are necessary for patients with cryptogenic liver abscesses, especially in eastern Asia, where gastrointestinal malignancies occur frequently. We found three patients with PLA and colon cancer in our study. Cryptogenic liver abscess has been mentioned in many studies, but the incidence of cryptogenic liver abscesses is difficult to calculate. Cryptogenicity is determined by how thoroughly one investigates to find the aetiology, e.g. Ultrasound scan to check for gallstones, EUS to check for microlithiasis, colonoscopy to check for polyps, cancer or diverticula, gastroscopy to check for gastric tumor, routine CT to check for appendicitis, cholecystitis, diverticulitis, colitis, or small bowel mass, or abscess formation *via* extension from a contiguous focus like the kidney. In addition, portal phlebitis, cholangitis, inflammatory bowel disease, infected liver cysts, and abdominal surgical infections. should be excluded. If one does not perform these tests, one will likely conclude to be cryptogenic.

Most patients had abnormal WBCs; elevated neutrophil/leukocyte ratios ratios, CRP, and PCT levels; and, abnormal liver function test results; however, the CRP and PCT levels were abnormal more often than the WBCs. We found elevated levels of CRP (in 99.1% of patients), PCT (in 83.5% of patients), neutrophil/leukocyte ratios (in 82.1% of patients), and WBCs (in 69.8% of patients). In our study, CRP was the most sensitive inflammatory response indicator. We used it to reveal the degree of inflammation caused by the infection and to evaluate the effect of the anti-infective treatment. CRP level reductions to a normal value can be used as an indicator of appropriateness of drug treatment cessation (24). Interestingly, we found three patients (2.8%) with low WBCs; a sign that can be present in patients with severe infections and that highlights the importance of diagnosing infections by means other than the WBC. During thrombocytopenia, CRP values >200 mg/L and PCT values >10 µg/L often indicate severe infection resulting in sepsis, hemodynamic disorders, organ dysfunction, and poor prognoses. We found 15 patients (14.2%) with shock out of the 106 patients in our cohort. Some of the patients with PLAs in our study had liver injuries; however, the degree of injury was mild and showed significant improvement after the implementation of infection control. In another study of 246 patients with PLA, the levels of CRP, PCT, and WBC showed no significant differences between the diabetes mellitus and the non-diabetes mellitus groups (15). In that paper, patients with diabetes mellitus showed significantly higher levels of ALP and γ-glutamyl transferase than their counterparts without diabetes mellitus.

Ultrasound, the first choice for the examination of liver abscesses because it is simple and accurate, and does not lead to radiation exposure, needs to be performed by experienced radiologists. The ultrasound examination is simple and noninvasive, and it displays the shape, size, quantity, location, liquefaction, and separation of abscesses in real time. Although the specificity of ultrasound can reach more than 85%, there are deviations in the observations of air cavities and the separation of the abscesses. The specificity of CT is higher than 95%, and liver abscesses with diameters of approximately 0.5 cm can be clearly detected. The “Petal sign” and “cluster sign” and other indirect signs of biliary tract problems can be diagnostic criteria for atypical liver abscesses during CT examinations. Some studies have shown that for *K. pneumoniae*-associated liver abscesses, the most frequent imaging manifestations are right lobe single abscesses in the liver parenchyma, with unclear boundaries, which are mostly solid and multilocular, and with air volumes in the abscess that are significantly larger than those usually seen in non-*K. pneumoniae* liver abscesses (25). The specificity and sensitivity of MRI scans are not as clear, but the “ring target sign” from MRI scans is an important clue for the diagnosis of a liver abscess.

In our cohort, the use of ultrasound-guided percutaneous aspiration followed by continuous catheter drainage along with parenteral antibiotic therapy showed a high success rate. Since 1953, administration of intravenous antibiotics after liver abscess puncture and drainage has become a classic method for the treatment of bacterial liver abscesses (26). The indications for puncture or catheter drainage are the following: (1) liver abscess with ineffective drug treatment or continuous increase of body temperature; (2) liver abscess with wall formation and liquefaction tending to mature; (3) abscess with a diameter of 3–5 cm that can be punctured and drained, and abscess with a diameter larger than 5 cm that can be drained with a tube; (4) liver abscess in patients with normal coagulation function and intolerance to operation. For abscesses larger than 10 cm, two drainage tubes can be placed from different angles to facilitate full drainage and flush the pus cavity as necessary. A meta-analysis reported success rates of 77.8% for percutaneous needle aspiration (PNA) and of 96.1% for percutaneous catheter drainage (PCD). PNA or PCD achieved clinical remission in a shorter time than antibiotic therapy alone. PCD is the first choice for the treatment of liver abscesses, not only because it is simple and cheap, but also because even for multiple liver abscesses that are difficult to treat, the treatment success rate is higher than 90% (27). Saleem Ahmed found that PCD is safe and sufficient even for patients with giant PLAs (28). We removed drainage tubes after the patient's laboratory examination and clinical performance results had returned to normal, the drainage fluid output was less than 5 ml/day, and imaging results had confirmed that the diameter of the pus cavity after drainage was smaller less than 2 cm. The study by Vishal G. Shelat followed a similar protocol (29).

In our study, the microbiological yield including both the pus and blood cultures was 72.6% (77/106), a number similar to that in a Singaporean study (30). The main isolates were *K. pneumoniae* (42 cases 54.5%) and *E. coli* (27 cases 35.1%). Over the past three decades, *K. pneumoniae* has emerged as the major pathogen and the single leading cause of PLAs in southern and eastern Asia, including in India, Korea, Singapore, Hong Kong, mainland China, and Taiwan. In a nationwide prospective study of PLAs in Korea, *K. pneumoniae* was the major etiological organism (7). Highly invasive *K. pneumoniae* strains possess genes responsible for a hypermucoviscosity phenotype associated with the serotypes K1 and K2. This highly invasive *K. pneumoniae* strains can cause intestinal colonization in healthy individuals that may lead to pathogenesis by opportunistic pathogens after the resulting microbiota compositional changes, especially the reduction in *Lactobacilli* abundance (31). When this occurs during bacterial translocation, pathogens can circulate to the liver through the portal vein, causing liver abscesses. After the gastrointestinal colonization by *K. pneumoniae* through environmental exposure or the fecal-to-oral transmission, the bacteria may cross the intestinal barrier to invade the liver (32). Asian populations may be predisposed to intestinal colonization by highly toxic *K. pneumoniae* strains, a fact that may explain the high prevalence of *K. pneumoniae* in patients with PLA in Asia. *E. coli* is the most common biliary liver abscess pathogen, accounting for 20%–35% of patients infected by *E.coli* alone (20), followed in frequency by *K. pneumoniae*. This may be the main reason for the differences in the etiologies between Asia and Europe. The main pathogen found in Europe was *E. coli*, accounting for 60% of PLAs. Vishal G. Shelat et al. found that in multimodal care settings, outcomes of *E. coli* PLA are comparable to those of *K. pneumoniae* PLAs (29). Moreover, multi-bacterial infections and multi-drug resistant bacteria are common, and their causes are associated with biliary tract diseases and abnormal bile duct anatomies. Abnormal endogenous intestinal flora compositions caused by infections are associated with the use of broad-spectrum antibiotics. Interestingly, Vishal G. Shelat et al. found that even though *K. pneumoniae* PLAs and culture-negative PLAs present demographic and clinical differences, their overall outcomes are equivalent, and they recommend treating culture-negative PLAs with empirical antibiotics targeting *K. pneumoniae* (30).

According to the recommended treatment scheme in the Sanford Guide to Antimicrobial Therapy of 2016, the first choice for PLA treatment is metronidazole combined with ceftriaxone, or cefoxitin, or piperacillin tazobactam, or ciprofloxacin, or levofloxacin; and, the alternative is metronidazole combined with imipenem/meropenem/donipenem. The patients in our cohort received third generation cephalosporins combined with metronidazole.

The optimal length of intravenous administration and subsequent oral maintenance remains unclear. Researchers in the USA and Chinese mainland have recommended that the intravenous administration be prolonged for 2–3 weeks, and the oral administration for 1–2 weeks. The course of treatment is determined by the response of the patient to treatment and should be adjusted according to the ultrasound findings, body temperature, and WBC counts (7, 8). If complicated with endophthalmitis, systemic venous and intravitreal anti-infection must be started, and ceftriaxone can be considered. For high-risk patients with advanced age, diabetes mellitus, intensive care unit (ICU) hospitalization, and catheterizations, carbapenem should be the first choice to treat suspected PLA infections. The empiric antibiotic treatment should be adjusted once the culture results become available.

There are limitations to our study. This was a retrospective analysis performed in a single center, some patients were excluded due to missing data, important and relevant details may not have been documented, and cases data were collected at the time of admission, and the actual changes in some indicators could not be judged by comparing baseline levels. However, our findings are based on a large number of cases and should be valuable to other investigators and clinicians.

## Conclusions

The common clinical manifestations of bacterial liver abscesses are fever and chills, right upper quadrant pain/epigastric discomfort, nausea and vomiting, and cough and sputum. Liver Doppler ultrasound was a conventional and effective method to diagnose PLAs in our study. *K. pneumoniae* is the most commonly isolated pathogen in PLAs. Percutaneous puncture under B-ultrasound guidance was the most commonly used treatment.

## Data Availability

The datasets presented in this study can be found in online repositories. The names of the repository/repositories and accession number(s) can be found in the article/**Supplementary Material**.
