# Supplementary figures and images for: Clinical characteristics and management of 106 patients with pyogenic liver abscess in a traditional Chinese hospital
Source: Front Surg. 2023 Jan 6;9:1041746. doi: 10.3389/fsurg.2022.1041746 (PMC9852512; doi:10.3389/fsurg.2022.1041746)

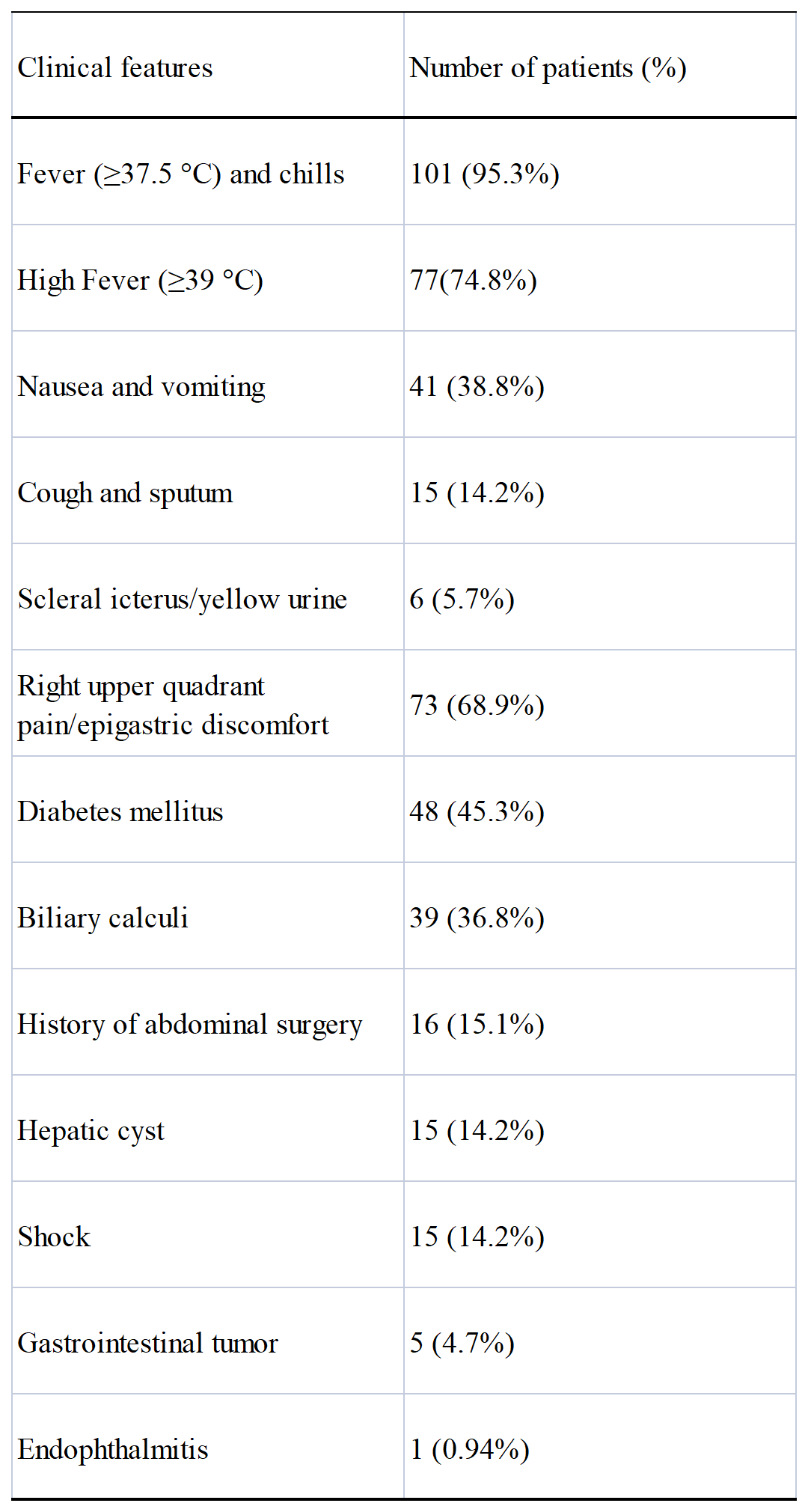

Supplement: Supplementary file 2 [file Image1.png]

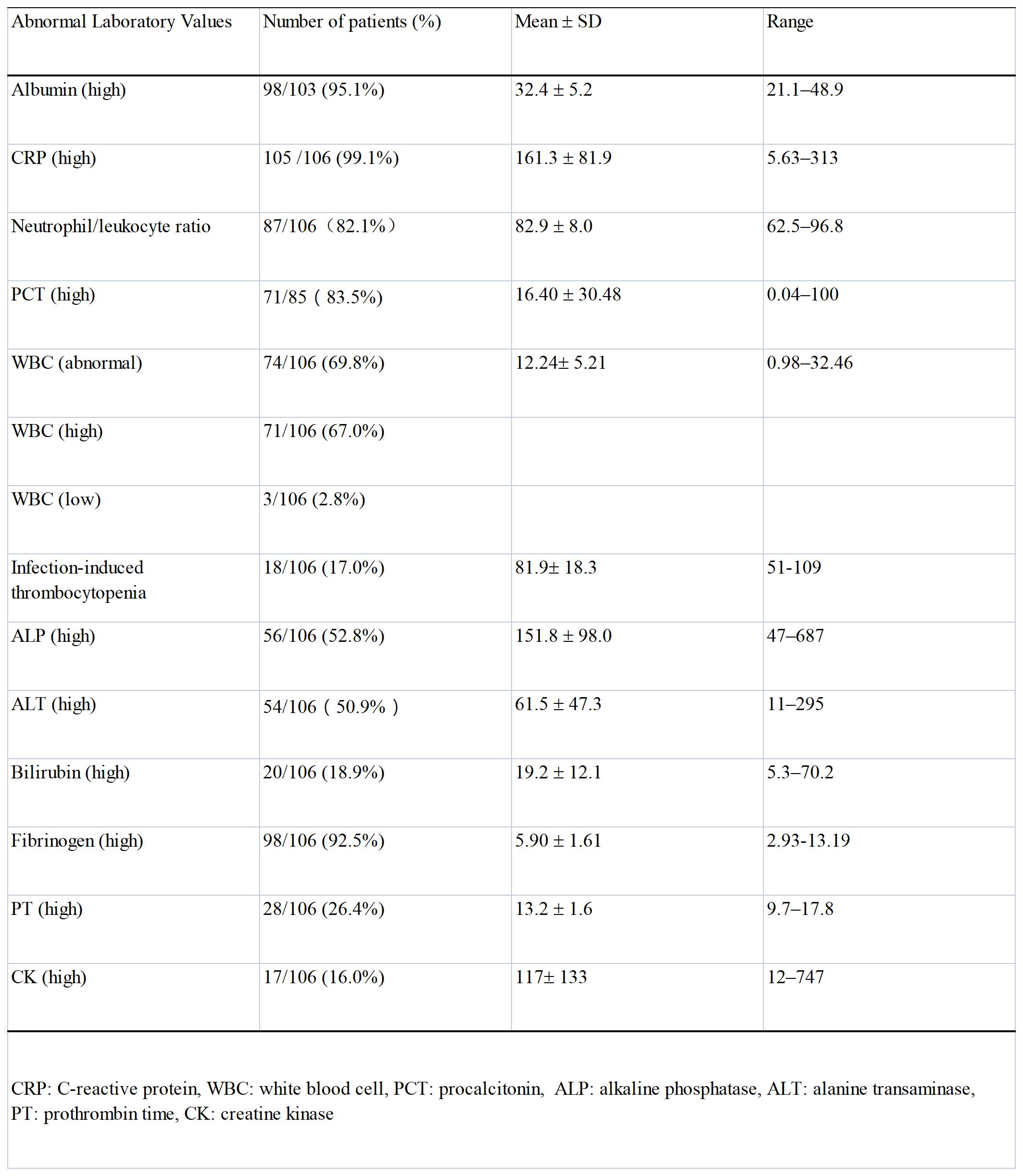

Supplement: Supplementary file 3 [file Image2.png]

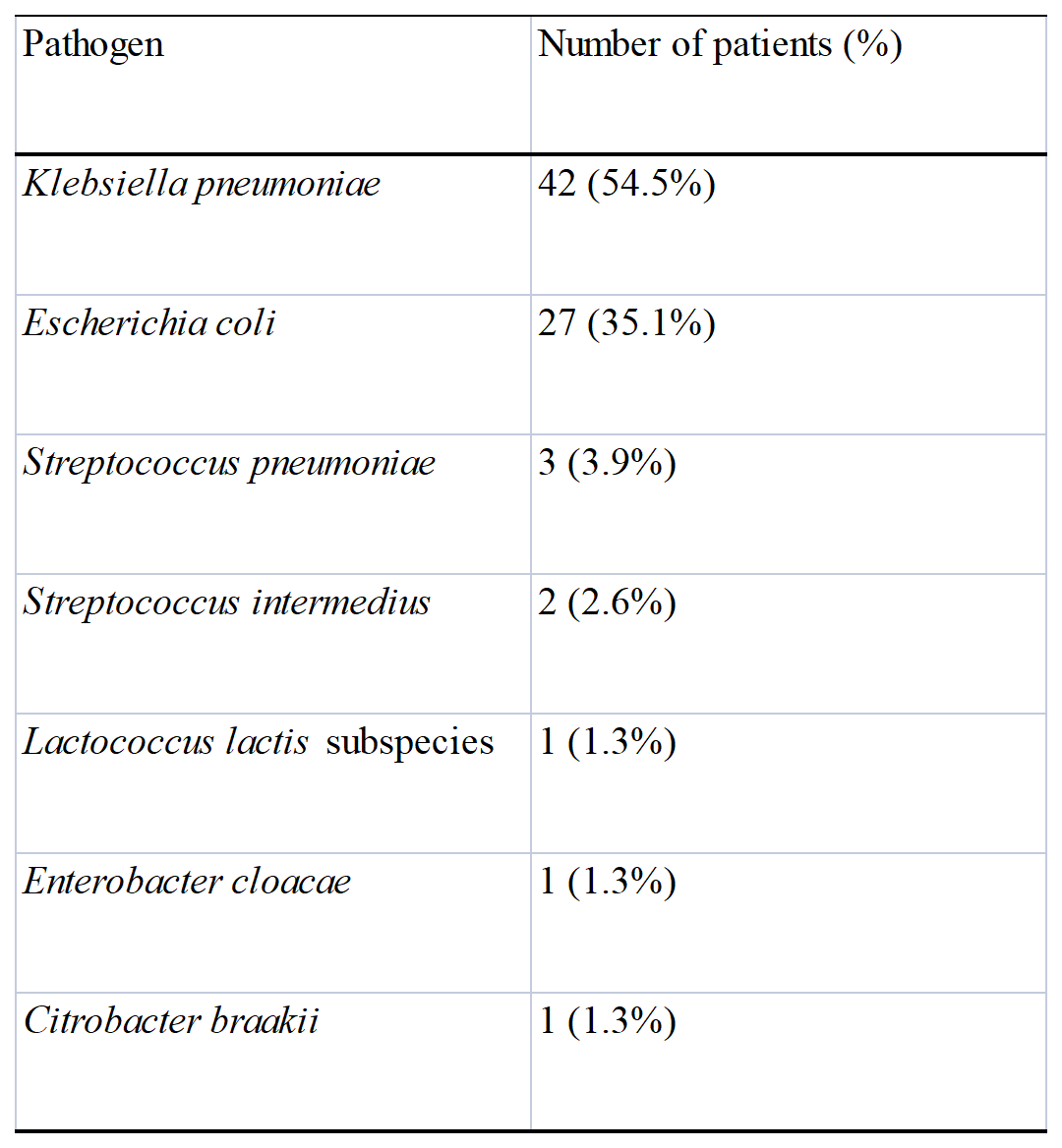

Supplement: Supplementary file 4 [file Image3.png]

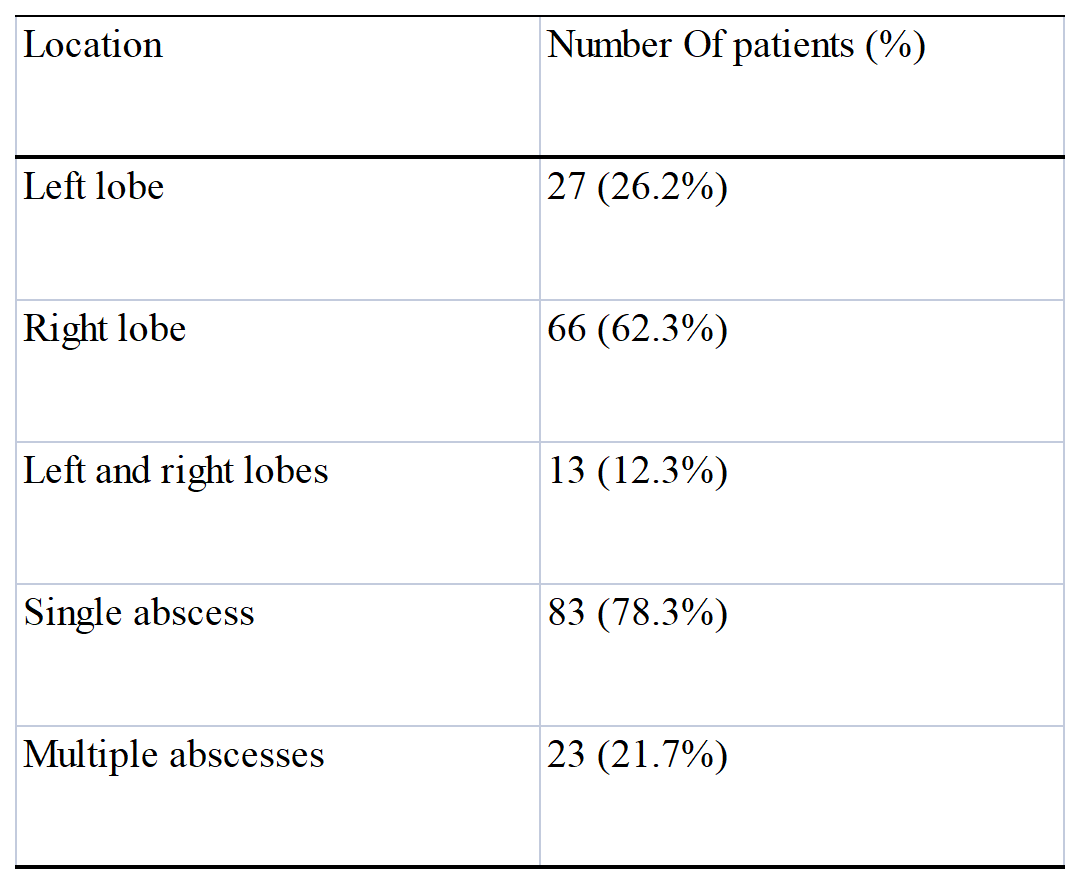

Supplement: Supplementary file 5 [file Image4.png]
